# Idiopathic intracranial hypertension: consensus guidelines on management

**DOI:** 10.1136/jnnp-2017-317440

**Published:** 2018-06-14

**Authors:** Susan P Mollan, Brendan Davies, Nick C Silver, Simon Shaw, Conor L Mallucci, Benjamin R Wakerley, Anita Krishnan, Swarupsinh V Chavda, Satheesh Ramalingam, Julie Edwards, Krystal Hemmings, Michelle Williamson, Michael A Burdon, Ghaniah Hassan-Smith, Kathleen Digre, Grant T Liu, Rigmor Højland Jensen, Alexandra J Sinclair

**Affiliations:** 1 Metabolic Neurology, Institute of Metabolism and Systems Research, University of Birmingham, Birmingham, UK; 2 Birmingham Neuro-Ophthalmology, Queen Elizabeth Hospital, Birmingham, UK; 3 Department of Neurology, University Hospital North Midlands NHS Trust, Stoke-on-Trent, UK; 4 Department of Neurology, The Walton Centre NHS Foundation Trust, Liverpool, UK; 5 Department of Neurosurgery, University Hospital North Midlands NHS Trust, Royal Stoke University Hospital, Stoke-on-Trent, UK; 6 Department of Neurosurgery, The Walton Centre NHS Foundation Trust, Liverpool, UK; 7 Department of Paediatric Neurosurgery, Alder Hey Children’s NHS Foundation Trust, Liverpool, UK; 8 Department of Neurology, Gloucestershire Hospitals NHS Foundation Trust, Cheltenham, UK; 9 Nuffield Department of Clinical Neurosciences, John Radcliffe Hospital, Oxford, UK; 10 Department of Neuroradiology, University Hospitals Birmingham, Queen Elizabeth Hospital, Birmingham, UK; 11 Department of Neurology, Sandwell and West Birmingham NHS Trust, Birmingham, UK; 12 Department of Neurology, University Hospitals Birmingham, Queen Elizabeth Hospital, Birmingham, UK; 13 IIH-UK charity, Tyne & Wear, UK; 14 Departments of Ophthalmology and Neurology, Moran Eye Center, University of Utah, Salt Lake City, Utah, USA; 15 Neuro-ophthalmology Services, Children’s Hospital of Philadelphia and Hospital of the University of Pennsylvania, Philadelphia, Pennsylvania, USA; 16 Danish Headache Center, Department of Neurology, Rigshospitalet-Glostrup, University of Copenhagen, Copenhagen, Denmark; 17 Centre for Endocrinology, Diabetes and Metabolism, Birmingham Health Partners, Birmingham, UK

**Keywords:** headache, benign intracran hyp, clinical neurology, neuroophthalmology, neurosurgery

## Abstract

The aim was to capture interdisciplinary expertise from a large group of clinicians, reflecting practice from across the UK and further, to inform subsequent development of a national consensus guidance for optimal management of idiopathic intracranial hypertension (IIH).

**Methods:**

Between September 2015 and October 2017, a specialist interest group including neurology, neurosurgery, neuroradiology, ophthalmology, nursing, primary care doctors and patient representatives met. An initial UK survey of attitudes and practice in IIH was sent to a wide group of physicians and surgeons who investigate and manage IIH regularly. A comprehensive systematic literature review was performed to assemble the foundations of the statements. An international panel along with four national professional bodies, namely the Association of British Neurologists, British Association for the Study of Headache, the Society of British Neurological Surgeons and the Royal College of Ophthalmologists critically reviewed the statements.

**Results:**

Over 20 questions were constructed: one based on the diagnostic principles for optimal investigation of papilloedema and 21 for the management of IIH. Three main principles were identified: (1) to treat the underlying disease; (2) to protect the vision; and (3) to minimise the headache morbidity. Statements presented provide insight to uncertainties in IIH where research opportunities exist.

**Conclusions:**

In collaboration with many different specialists, professions and patient representatives, we have developed guidance statements for the investigation and management of adult IIH.

## Scope

This is a consensus document to provide practical information for best practice in uniform investigation and treatment strategies based on current literature and opinion from a specialist interest group (SIG) for adult idiopathic intracranial hypertension (IIH). This should increase awareness of IIH among clinicians and improve outcomes for patients.

The target audience for this statement includes neurologists, ophthalmologists, neurosurgeons, radiologists, emergency medicine specialists, physicians, ear nose and throat specialists and other clinicians who investigate and manage IIH. It also contains information that will be of interest to those in primary care and other healthcare professionals.

The increasing economic burden of IIH has been highlighted by a number of groups.^1 2^ Clear guidance will help educate the attending doctors to manage these patients appropriately. This will help reduce the repeat unsolicited emergency hospital attendances and reduce IIH-related disability. There are a number of ongoing clinical trials in IIH (https://www.clinicaltrials.gov/) and as evidence for medical and surgical management evolves in IIH this document will require timely updates.

## Background

IIH occurs predominantly in women and although the underlying pathogenesis is not fully understood, it has a striking association with obesity.^3^ The combination of raised intracranial pressure, without hydrocephalus or mass lesion, normal cerebrospinal fluid (CSF) composition and where no underlying aetiology is found are accepted criteria for the diagnosis of IIH.^4^ The overall age-adjusted and gender-adjusted annual incidence is increasing and was reported to be 2.4 per 100 000 within the last decade (2002–2014).^5^

**Figure 1:**
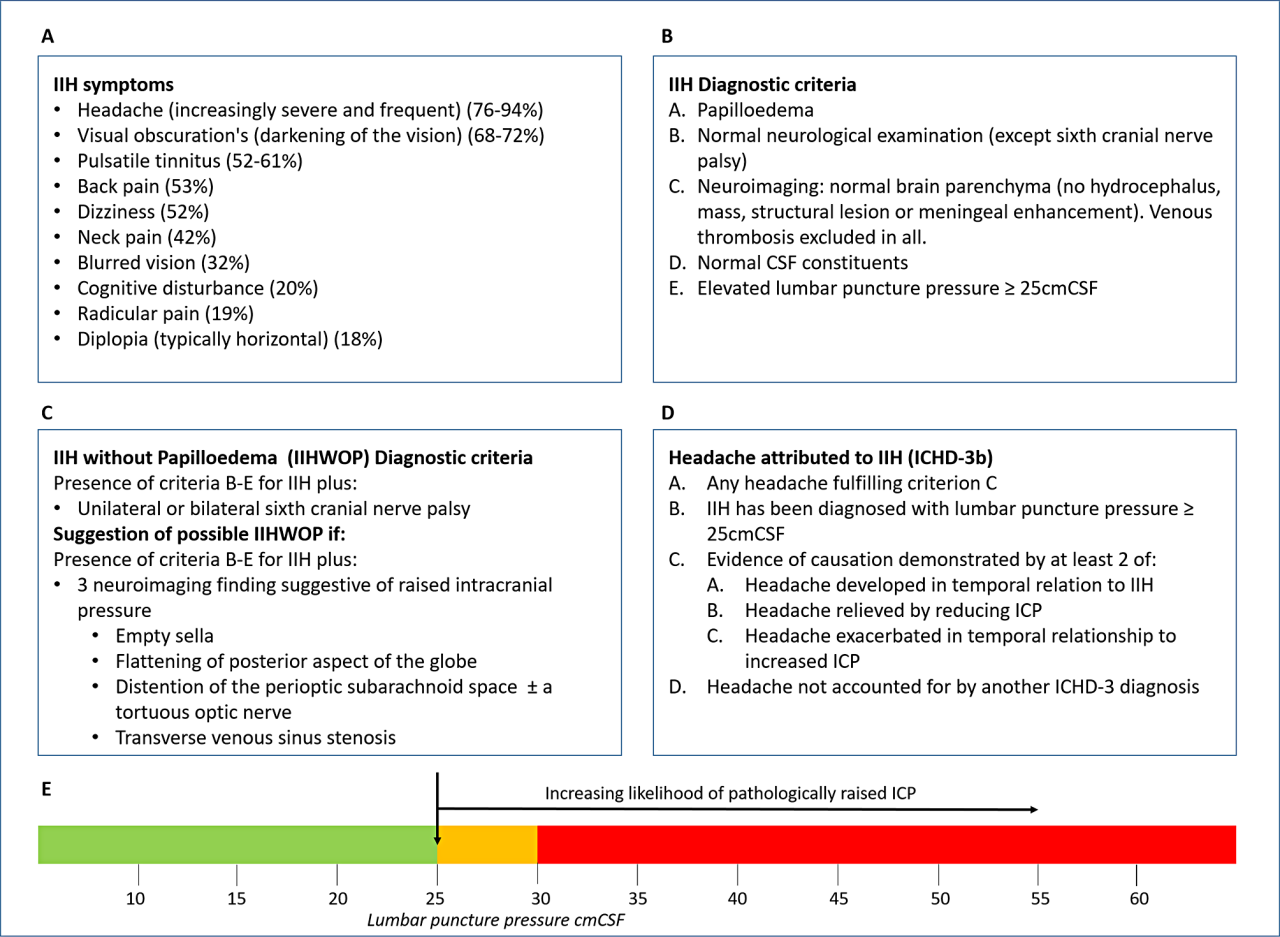
Consensus in diagnosing IIH. (A) Frequency of IIH symptoms reported, adapted from Markey *et al.* 3 (B) IIH diagnostic criteria, adapted from Friedman *et al*.^4^ (C) IIHWOP diagnostic criteria, adapted from Friedman *et al*.^4^ (D) Headache attributed to IIH, as described by the International Classification of Headache Disorders, 3rd edition (beta version) (ICHD-3 beta).^6^ (E) Line figure detailing the consensus of the interpretation of LP opening pressure. Uncertainty: it needs to be recognised that this is a single LP OP measurement; and after raised ICP what is then a normal ICP for this population on repeat LP readings is unknown. CSF, cerebrospinal fluid; IIH, idiopathic intracranial hypertension; LP, lumboperitoneal.

The majority of patients presenting with IIH have symptoms that include a headache that is progressively more severe and frequent, as defined by International Classification of Headache Disorders, 3rd edition (ICHD-3) (figure 1).^6^ The headache phenotype is highly variable and may mimic other primary headache disorders. Other symptoms may include transient visual obscurations (unilateral or bilateral darkening of the vision typically seconds), pulsatile tinnitus, back pain, dizziness, neck pain, visual blurring, cognitive disturbances, radicular pain and typically horizontal diplopia (figure 1A)^3^: none of which are pathognomonic for IIH.^7^ Investigation and management depends on symptoms and signs and requires an interdisciplinary team approach.

For the individual patient, some can have permanent visual loss.^8^ Chronic headache significantly impacts quality of life^9 10^ with over half of patients with IIH reporting ongoing headaches at 12 months.^11^

Clinical uncertainty exists, and IIH can be misdiagnosed.^12^ The 2015 Cochrane review has concluded that there is lack of evidence to guide pharmacological treatment in IIH.^13^ Randomised clinical trials are currently infrequent in this field due to the rarity of the disease, the lack of understanding of the underlying pathological mechanisms and limited disease-modifying therapies.

## Methods

An SIG was formed, including neurology, neurosurgery, neuroradiology, ophthalmology, nursing, primary care doctors and patient representatives. All clinicians had expertise in managing IIH. An initial UK survey of attitudes and practice in IIH was sent to a wide group of consultants who investigate and manage IIH regularly: these included neurology, neurosurgery, neuro-ophthalmology and neuroradiology. A comprehensive systemic literature review was performed to assemble the foundations of the statements. Rigorous controlled data are sparse in IIH, and therefore, a consensus-based guide is presented. Questions were formulated (table 1). An anonymous modified Delphi process was used to obtain consensus on guidance statements. All statements below obtained consensus of 75% or above from the SIG and wider Delphi group. A completed AGREE statement is found as supplementary data (online supplementary appendix 1).

10.1136/jnnp-2017-317440.supp2 Supplementary file 2

An international panel of experts in IIH (RHJ, GTL and KD) reviewed the document and a wider consultation was made with professional bodies namely the Association of British Neurologists (ABN), the Society of British Neurological Surgeons (SBNS), the Royal College of Ophthalmologists (RCOphth) and the British Association for the Study of Headache (BASH). Where there was disagreement in statement recommendations, these were debated within the SIG, and wording was altered accordingly.

Specifically, to improve local outcomes for patients with IIH, audit recommendations are enclosed (online supplementary appendix 2). This document will need to be revised regularly as new evidence emerges in the field of IIH. Definitions used in the guidance are presented in table 2.^14–17^

10.1136/jnnp-2017-317440.supp3 Supplementary file 3

**Table 1:** Questions formulated by the ABN IIH SIG on the diagnosis and management of IIH

Question number	
	Diagnostic principles
1	*How should papilloedema be investigated?*
	Management principles Principle one: treat the underlying disease
2	*What is the best way to modify the underlying disease to induce remission?*
	Principle two: protect the vision
3	*How should IIH be treated when there is imminent risk of visual loss?*
4	*What is currently the best surgical procedure for visual loss in IIH?*
5	*What other surgical procedures are performed for visual loss in IIH?*
6	*What is the current role of neurovascular stenting in acute IIH to prevent loss of vision?*
7	*What is the role of serial lumbar punctures in IIH?*
8	*What is the best drug treatment for IIH symptoms?*
9	*How should acetazolamide be prescribed?*
10	*Are there other drugs that are helpful in IIH?*
	Principle three: manage the headache
11	*What is the best way to manage headaches in **newly diagnosed** IIH?* (figure 4)
12	*What is the best approach to long-term headache management in IIH?*
13	*What therapeutic strategies are useful for headache in IIH?*
14	*How should medication overuse headache be approached?*
15	*Should CSF diversion surgery be used in patients with IIH with headache alone?*
16	*Should neurovascular stenting be used in patients with IIH with headache alone?*
17	*How should an acute exacerbation of headache be investigated in those who are already shunted?*
18	*How should an acute exacerbation of headache be treated in those who are already shunted?*
	Clinical care and managing IIH in pregnancy
19	*Are there any other chronic problems that need to be addressed in IIH?*
20	*What advice should be given regarding drug treatments in the pregnant patient with IIH?*
21	*What additional considerations for management are there in the pregnant patient with IIH?*
	IIHWOP
22	*How should IIHWOP be managed?*
	Follow-up and monitoring of IIH
23	*How should we follow-up and monitor these patients?*

ABN, Association of British Neurologists; CSF, cerebrospinal fluid; IIH, idiopathic intracranial hypertension; IIHWOP, IIH without papilloedema; SIG, specialist interest group.

**Table 2:** Definitions of the terms used in the guidance

Term	Definition
Adult	All patients above the age of 16 years old for the purpose of this statement.
Idiopathic intracranial hypertension (IIH)	Patients with raised ICP of unknown aetiology fulfilling the criteria set out in figure 1.
Fulminant IIH	Patients meeting the criteria for a precipitous decline in visual function within 4 weeks of diagnosis of IIH.^14^
Typical IIH	Patients who are female, of childbearing age and who have a body mass index (BMI) greater than 30 kg/m^2^.
Atypical IIH	Patients who are not female, or not of childbearing age or who have a BMI below 30 kg/m^2^. These patients require more in-depth investigation to ensure no other underlying causes (table 2).^15 25^
IIH without papilloedema	A rare subtype of IIH^16 17^ and is seen in patients who meet all the criteria of definite IIH,^4^ seen in figure 1, in the absence of papilloedema. The criteria have highlighted the importance of a pressure greater than 25 cm CSF and the necessity for additional features, which suggest pathologically raised ICP. Features such as sixth nerve palsy and MRI imaging features indicating raised ICP should be sought (box 1).
IIH in ocular remission	Patients that have been diagnosed as IIH, and the papilloedema has resolved. These patients may have ongoing morbidity from headache, but their vision is no longer at risk while there is no papilloedema.
Experienced clinician	Refers to any clinician, in the context of this guidance, who has confidence in their own experience of managing IIH.

Box 1 **Typical neuroimaging features found in raised intracranial pressure^19–22^**

Neuroimaging features of raised ICP:

- empty sella
- partially empty sella/decreased pituitary height
- increased tortuosity of optic nerve
- enlarged optic nerve sheath (perioptic subarachnoid space)
- flattened posterior globe/sclera
- intraocular protrusion of optic nerve head
- attenuation of the cerebrovenous sinuses including bilateral transverse sinus stenosis or stenosis of a dominant transverse sinus.

Note: Enhancement with IV contrast of the optic nerve sheath has been reported. Additionally, ventricle size in IIH is typically normal however many reports consider the ventricles to be slit-like.

## Diagnostic principles

For optimal investigation of patients with papilloedema, there must be clear communication between clinicians for seamless joint investigation between the various specialities. The aims of investigations of papilloedema are to:

1. find any underlying treatable cause in a timely manner
2. protect the vision and ensure timely re-examination when vision is at risk
3. enable onward care of the patient with the input from the most appropriate experienced clinician.

### Q1 How should papilloedema be investigated? (figure 2)

- *Blood pressure* must be measured to exclude malignant hypertension, as defined as a diastolic blood pressure greater than or equal to 120 mm Hg or systolic blood pressure greater than or equal to 180 mm Hg.^18^
- *Ophthalmology examination*: all patients should have papilloedema confirmed and an assessment made of the imminent risk to their visual function. The following should be recorded in the presence of papilloedema:
  - visual acuity
  - pupil examination
  - intraocular pressure (to exclude hypotony, a rare cause for disc swelling)
  - formal visual field test (perimetry)
  - dilated fundal examination to grade the severity of the papilloedema and exclude ocular causes for disc swelling.

Where possible, document the fundus picture with drawings and document key findings on the optic nerve head (hyperaemia, haemorrhages, cotton wool spots, obscuration of the vessels and so on). Photographs and/or optical coherence tomography (OCT) imaging are useful. Where visual function is found to be threatened, regular ophthalmic examination must occur because this will influence timely management (see *23 How should we follow-up and monitor these patients?* in table 3).

**Figure 2:**
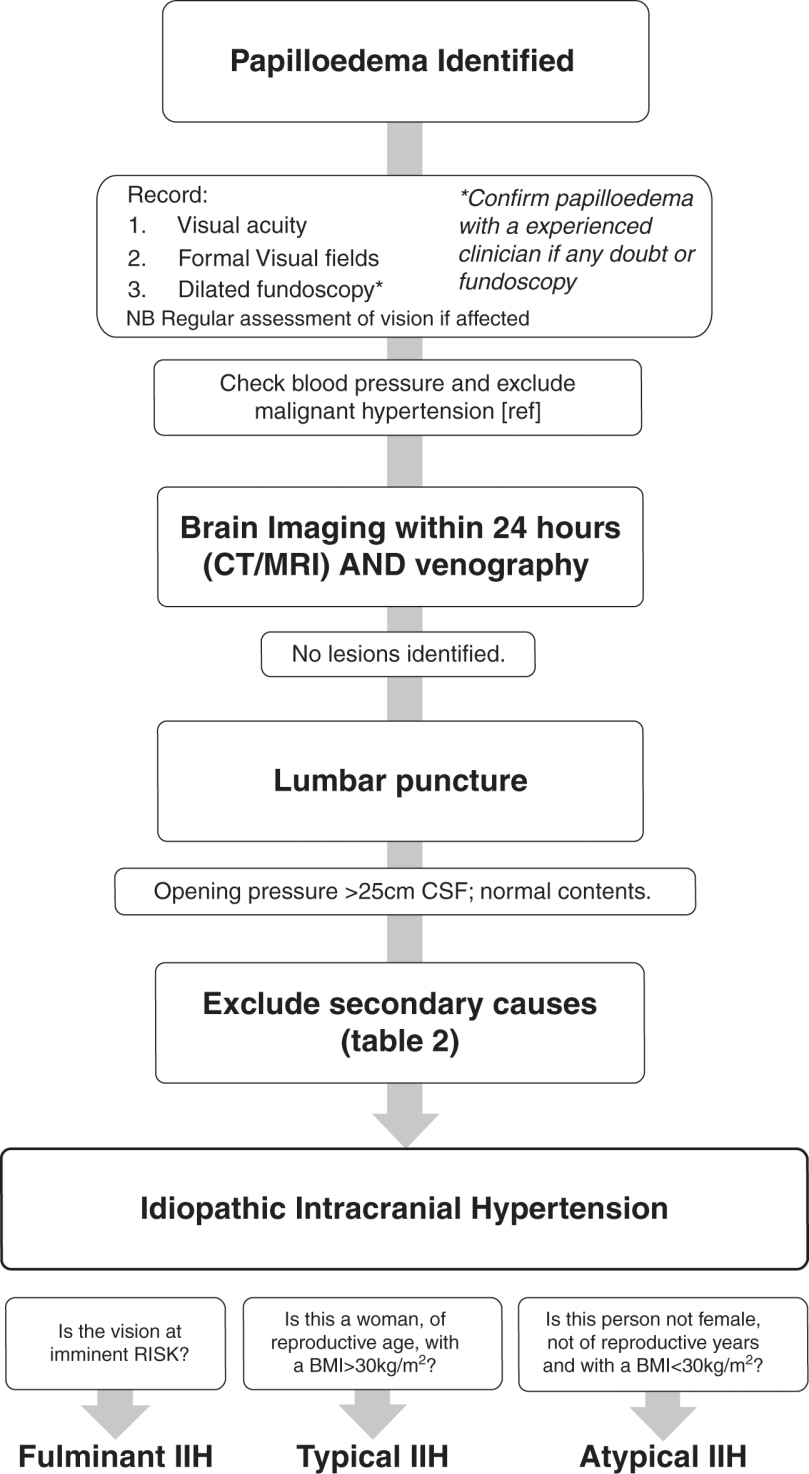
A flow diagram of investigation of papilloedema. BMI, body mass index; IIH, idiopathic intracranial hypertension.

**Table 3:** Associations that have been reported as causing raised Intracranial pressure^15 25^

Haematological	Anaemia Polycythaemia vera
Obstruction to venous drainage	Cerebral venous sinus thrombosis
	Jugular vein thrombosis
	Superior vena cava syndrome
	Jugular vein ligation following bilateral radical neck dissection
	Increased right heart pressure
	Arteriovenous fistulas
	Previous infection or subarachnoid haemorrhage causing decreased CSF absorption
Medications	Fluoroquinolones
	Tetracycline class antibiotics
	Corticosteroid withdrawal
	Danazol
	Vitamin A derivatives (including isotretinoin and all-transretinoic acid)
	Levothyroxine
	Nalidixic acid
	Tamoxifen
	Ciclosporin
	Levonorgestrel impant
	Lithium
	Growth hormone
	Indomethacin
	Cimetidine
Systemic disorders	Chronic kidney disease/renal failure
	Obstructive sleep apnoea syndrome
	Chronic obstructive pulmonary disease
	Systemic lupus erythematosus
	Psittacosis
Endocrine	Addison’s disease
	Adrenal insufficiency
	Cushing’s syndrome
	Hypoparathyroidism
	Hypothyroidism
	Hyperthyroidism
Syndromic	Down syndrome
	Craniosynostosis
	Turner syndrome

#### Uncertainty

Where there is diagnostic uncertainty regarding papilloedema see the differential diagnosis of papilloedema and pseudopapilloedema in supplementary table 1, an experienced clinician should be consulted early before invasive tests are performed.

10.1136/jnnp-2017-317440.supp1 Supplementary file 1

- *Neurological examination*
  - Record cranial nerve examination. Where IIH is suspected, typically there should be no cranial nerve involvement other than sixth cranial nerve palsy/palsies.
  - Should other cranial nerves and/or other pathological findings be involved, an alternative diagnosis should be considered.
- *Neuroimaging*
  - Urgent MRI brain within 24 hours; if unavailable within 24 hours, then urgent CT brain with subsequent MRI brain if no lesion identified.
  - There should be no evidence of hydrocephalus, mass, structural, vascular lesion and no abnormal meningeal enhancement.^4^
  - CT or MR venography is mandatory to exclude cerebral sinus thrombosis within 24 hours.
  - Characteristics of raised intracranial pressure may be seen on neuroimaging (box 1); these are not pathognomonic of IIH.^19–22^

#### Uncertainty

We recognise the difficulties in the interpretation of cerebral venography. Where there is diagnostic uncertainty regarding interpretation of the venogram findings, an experienced radiologist should be consulted.

- *Lumbar puncture*
  - Following normal imaging, all patients with papilloedema should have a lumbar puncture to check opening pressure and ensure contents are normal.
  - The lumboperitoneal (LP) opening pressure should be measured in the lateral decubitus position.^4^ Following needle insertion into the CSF space, the pressure recording should occur with the patient relaxed and the legs extended. The CSF level should be allowed to settle before taking the reading.
  - The CSF analysis should be tailored to the presentation but should at a minimum include CSF protein, glucose and cell count.
  - A clear explanation of the LP should be given to patients to reduce fear and anxiety about the procedure.
  - Where difficulty exists in performing the LP, the length of the procedure should be balanced by the comfort of the individual patient.
  - Should the LP not be successful, a guided LP could then be considered (ultrasound or X-ray).^23 24^
  - The diagnostic criteria mandate a cut-off opening pressure of >25 cm CSF for diagnosing IIH.^4^
  - The LP opening pressure should not be interpreted in isolation when diagnosing IIH.

#### Uncertainties

Clinicians debate the absolute LP opening value of 25 cm CSF as diagnostic of IIH. This was recognised by Friedman and colleagues.^4^ Below the cut-off of 25 cm CSF, there are reservations as to the likelihood of diagnosing IIH. As highlighted in figure 1E, the SIG clinicians’ opinions are that there is an increasing likelihood of the significance of LP OP measurement, as it rises. The LP OP is a single measurement, and it is widely recognised that there is a diurnal and wide variation in CSF pressure.

Where the LP OP does not fit the clinical picture, it should be interpreted with caution. A repeat LP may be considered or intracranial ICP monitoring could be considered. There is no current evidence to dictate how much CSF is recommended to be drained or what the closing pressure should be. 

- *Exclusion of all other secondary causes of raised ICP*
  - All should have a careful history taken to exclude any possible secondary causes that have previously been linked to raised intracranial hypertension (table 2), although the causal link with IIH and a number of diseases and medications is not clear.^15 25^
  - All patients should have a full blood count performed to exclude anaemia.^26 27^
  - Where patients are deemed to be atypical (table 1), other additional blood tests may be considered to exclude secondary causes.
  - Where patients are deemed to be atypical (table 1), additional neuroimaging might be considered. These may include more proximal imaging of the neck vasculature to exclude internal jugular obstruction.

#### Uncertainty

In those with IIH, there is no clear evidence of a contraindication for using medications (including the oral contraceptive) that have been previously been reported to be casually associated with secondary pseudotumour.

Where uncertainty exists, patients who have atypical aspects could be referred for an opinion from a experienced clinician familiar with IIH.

## Management principles

**Figure 3:**
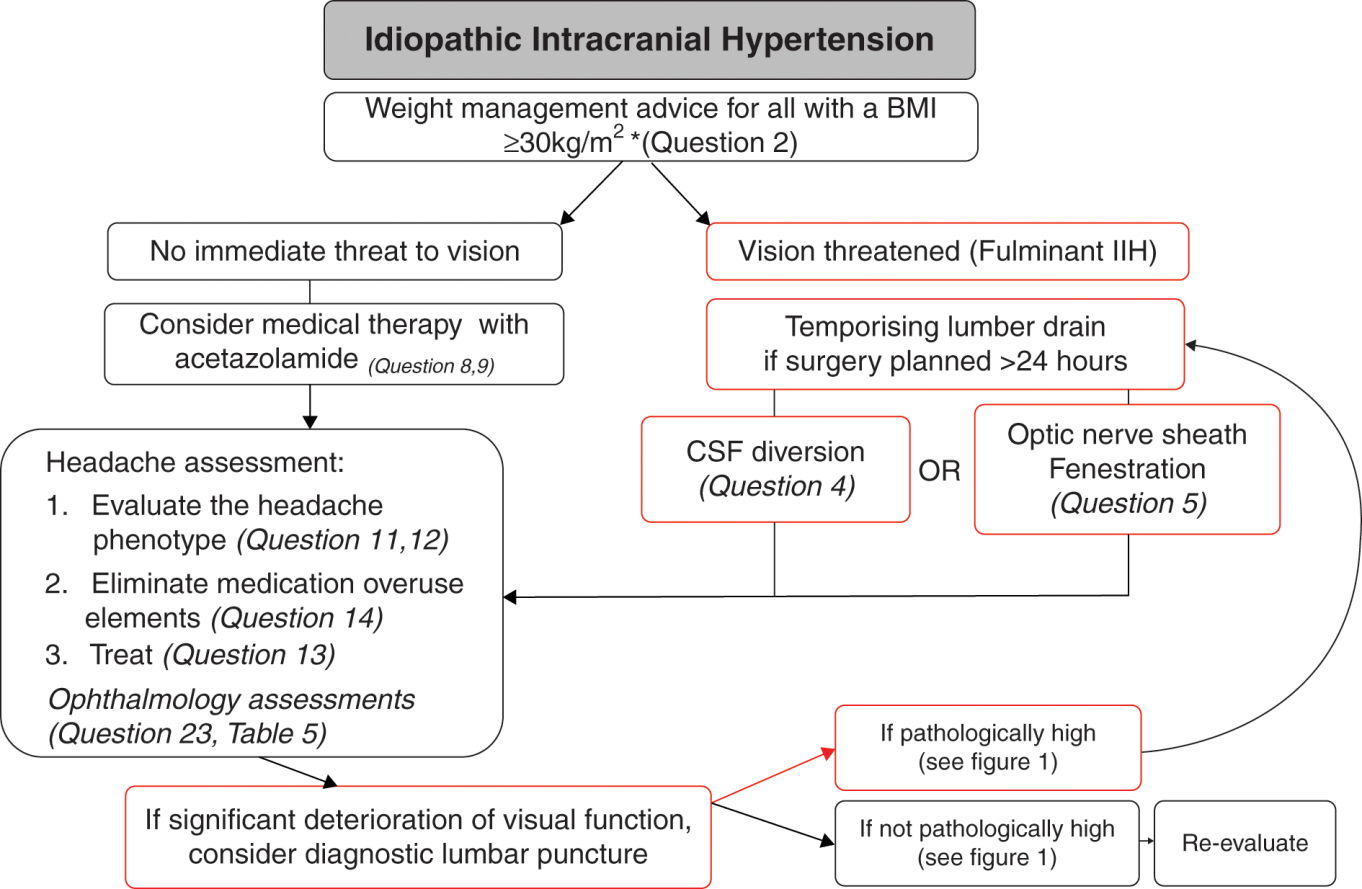
Management flow chart of diagnosed IIH. BMI, body mass index; CSF, cerebrospinal fluid; IIH, idiopathic intracranial hypertension.

For optimal management of patients with IIH, there must be clear communication between clinicians for seamless joint care between the various specialties (figure 3). Weight loss reduces ICP and has been shown to be effective in improving papilloedema and headaches.^28^ The main principles of management of IIH are:

1. to treat the underlying disease
2. to protect the vision
3. to minimise the headache morbidity.

Twenty-three questions were formulated to cover the three principle domains of management in IIH (table 1).

### Primary principle for IIH management: modify the underlying disease through weight loss

#### Q2 What is the best way to modify the disease to induce remission?

Weight loss is the only disease-modifying therapy in typical IIH.^28^

- Once definite IIH is diagnosed, all patients with a BMI >30 kg/m^2^ should be counselled about weight management at the earliest opportunity. This should be done with sensitivity.
- The amount of weight loss required to put the disease into remission is not known. It is noted that in the year preceding a diagnosis of IIH is associated with 5%–15% wt gain,^29^ and up to 15% of weight loss was required to put IIH into remission in one cohort.^28^
- Patients should be referred to a community weight management programme or a hospital-based weight programme.

##### Uncertainty

Maintained weight loss is difficult to achieve, and the optimal approach to achieving long-term weight management has not yet been clearly established.^30 31^ If weight loss cannot be achieved by the patients themselves, the first step would be professional help through a structured diet. There is an increasing role for bariatric surgery for sustained weight loss,^31 32^ and for use in IIH, more prospective controlled evidence is required.^2 33 34^ For those who are not obese secondary causes should be revisited (box 1), and the role of weight gain/loss remains uncertain.

### Second principle for IIH management: protect the vision

#### Q3 How should IIH be treated when there is imminent risk of visual loss?

- Where there is evidence of declining visual function, the acute management to preserve vision is surgical.
- A temporising measure of a lumbar drain could be useful to protect the vision while planning urgent surgical treatment.
- There is evidence that many of the surgical procedures, such as CSF diversion and optic nerve sheath fenestration (ONSF), work well in the short term.^35^ While they are working, the underlying disease should be modified with weight loss (see *1. What is the best way to modify the disease to induce remission?* in table 3).

##### Uncertainty

In the absence of high class evidence, we do not recommend the use of corticosteroids for fulminant IIH at this time, and indeed a prolonged treatment course of corticosteroids would not be recommended due to weight gain.

#### Q4 What is currently the best surgical procedure for visual loss in IIH?

- In the UK, the preferred surgical procedure is neurosurgical CSF diversion (see *5. What other surgical procedures are performed for visual loss IIH?*).
- Where possible. it should be performed by a experienced clinician with an interest in CSF disorders.
- Ventriculoperitoneal (VP) should be the preferred CSF diversion procedure for visual deterioration in IIH, due to lower reported revisions per patient.^2^
- An LP shunt could also be used.
- It is best practice to use neuronavigation to place VP shunts.
- All patients in the UK should be counselled that they should inform the Driver and Vehicle Licensing Agency following VP shunt placement.
- Adjustable valves with antigravity or antisiphon devices should be considered for use to reduce the risk of low pressure headaches.

##### Uncertainty

The literature pertaining to shunt type is observational and mainly case series based. Complications of shunts include abdominal pain, shunt obstruction, migration and infection, low pressure headaches, subdural haematoma and tonsillar herniation.^36 37^ There is a low, but present, mortality rate with CSF diversion; these figures do not come from IIH cohorts.

#### Q5 What other surgical procedures are performed for visual loss in IIH?

ONSF is performed more frequently in Europe and the USA and rarely in the UK. ONSF is reported to have less complications than CSF diversion, and there have been no reports of mortalities in the literature. The reported temporary adverse effects include double vision, ansiocoria and optic nerve head haemorrhages. Very rarely more permanent sequelae that include branch and central retinal artery occlusions have been reported. Some consider ONSF as the first treatment step in malignant fulminant cases and eventually also for those with asymmetric papilloedema causing visual loss in one eye.^38^ If this procedure fails, then the more invasive CSF diversion can be considered. ONSF should be performed by an experienced clinician trained in this technique.

##### Uncertainty

The literature is observational and mainly case series based.^38 39^ Treatment failure rates include worsening in vision after a period of stabilisation in 34% of patients at 1 year and 45% at 3 years. There is also failure to improve headache in one third to one-half.^39^

#### Q6 What is the current role of neurovascular stenting in acute IIH to prevent loss of vision?

Improvements in venography imaging now detail that many with IIH have anatomical abnormalities of the cerebral venous sinus system. These include stenosis of the dominant or both transverse sinus. The stenosis may result from intrinsic dural sinus anatomy or extrinsic compression by the increased intracranial pressure and reducing ICP can led to resolution of stenosis. The degree of stenosis does not appear to uniformly correlate with intracranial pressure or visual loss.^40^ Neurovascular stenting has been reported, in a number of series, to lead to an improvement in symptoms of intracranial hypertension. Complications of the procedure include a short-lived ipsilateral headache in many, stent-adjacent stenosis that require retreatment in a third and in rare cases vessel perforation leading to acute subdural haematoma, stent migration and thrombosis.

- The role of neurovascular stenting in IIH is not yet established.
- Long-term antithrombotic therapy is required for longer than 6 months following neurovascular stenting treatment.

##### Uncertainty

The literature is observational and mainly case series based, and there is no long-term data regarding efficacy and safety. The role of neurovascular stenting in IIH to preserve rapidly deteriorating vision is not yet established, as there is a lack of quality data in this area. It may be useful for highly selected patients with IIH with venous sinus stenosis with an elevated pressure gradient and elevated ICP in whom traditional therapies have not worked.^40 41^

#### Q7 What is the role of serial lumbar punctures in IIH?

The relief from a LP is typically short lived as CSF is secreted from the choroid plexus at a rate of 25 mL/hour and consequently the volume removed in a so-called therapeutic tap is rapidly replaced.^42^

- Serial lumbar punctures are not recommended for management of IIH.
- Despite the relief of headache in nearly three quarters of patients,^43^ LPs are associated with significant anxiety in many patients and can led to acute and chronic back pain in some patients.^7 43^

#### Q8 What is the best drug treatment for IIH symptoms?

The current Cochrane review on IIH management reported on the use of acetazolamide, a carbonic anhydrase inhibitor, in IIH. It concluded: ‘the two included RCTs showed modest benefits for acetazolamide for some outcomes, there is insufficient evidence to recommend or reject the efficacy of this intervention, or any other treatments currently available, for treating people with IIH’.^13^

The two studies included in this review were:

- The IIH Treatment Trial^44^ reported the use of acetazolamide with a low-sodium weight-reduction diet compared with diet alone resulted in modest improvement in visual field function in patients with mild visual loss. The IIHTT also reported improved quality of life outcomes at 6 months with acetazolamide.^45^
- Ball *et al* 46 failed to show a treatment effect. Importantly, 48% discontinued acetazolamide due to adverse effects.

- Acetazolamide could be prescribed for those with IIH symptoms.
- All females with IIH when commencing any new medical therapy (whether IIH specific or headache related) must be counselled regarding side effects and potential teratogenetic risks (see *21. What additional considerations for management are there in the pregnant patient with IIH?* in table 3).
- Drug therapies may need to be altered due to adverse side effects, lack of efficacy, possible potential teratogenic effects in pregnancy or patient preference.

##### Uncertainty

In view of the limited evidence as reported by the 2015 Cochrane review^13^ and the side effect profile, not all clinicians in the UK prescribe acetazolamide for IIH.

#### Q9 How should acetazolamide be prescribed?

- The IIHTT used a maximal dose of 4 g daily, with 44% of participants achieving 4 g/day, and the majority tolerating 1 g/day.^47^ Ball *et al* 46 identified that 48% discontinued at mean doses of 1.5 g due to side effects.
- A popular starting dose of acetazolamide is 250–500 mg twice a day, with the majority of clinicians titrating the daily dose up.
- Patients should be warned of the adverse side effects of acetazolamide that are well recognised and include increased risk of diarrhoea, dysgeusia, fatigue, nausea, paraesthesia, tinnitus, vomiting, depression and rarely renal stones.
- There is no consensus over the use of normal release and modified release acetazolamide.

##### Uncertainties

The optimal dose of acetazolamide is not established. The licencing information regarding acetazolamide recommends periodic monitoring of serum electrolytes; however, there is no consensus on the timing of monitoring.

#### Q10 Are there other drugs that are helpful in IIH?

Topiramate has carbonic anhydrase activity and can suppress appetite. It has been compared with acetazolamide in an uncontrolled open label study for IIH.^48^ Participants were alternately assigned to the treatments, not randomly, and there was no placebo control group. There is evidence of efficacy of topiramate in treating migraine.^49^

- There may be a role for topiramate in IIH with weekly dose escalation from 25 mg to 50 mg bd.
- Where topiramate is prescribed, women must be informed that it can reduce the efficacy of the contraceptive pill/oral contraceptives and other hormonal contraceptives.
- When topiramate is prescribed, women must be counselled regarding side effects (including depression and cognitive slowing) and potential teratogenetic risks.

##### Uncertainties

The role of other diuretics such as furosemide, amiloride and coamilofruse are not certain but are used by some as alternative therapies.

### Third principle of IIH management: reduce headache disability

Raised ICP can drive headaches, which may be very severe at presentation.^11^ Despite significant headache morbidity in IIH, there are no randomised controlled trials to guide headache management in IIH.

#### Q11 What is the best way to manage headaches in newly diagnosed IIH?

- Patients must be informed, at the earliest opportunity, of the potential issues of painkiller overuse that can lead to medication overuse headache (use of simple analgesics on more than 15 days per month or opioids, combined preparations or triptan medication on greater than 10 days per month for more than 3 months).^6^
- Short-term painkillers may be helpful in the first few weeks following diagnosis. These could include non-steroidal anti-inflammatory drugs (NSAIDs) or paracetamol. Indomethacin may have some advantage due to its effect of reducing ICP.^50^ Caution is required with potential side effects of NSAIDs, and gastric protection may be needed.
- Opioids should not be prescribed for headaches.^51^
- Greater occipital nerve blocks maybe considered helpful by some, but there is a lack of evidence and consensus.
- Acetazolamide has not been shown to be effective for the treatment of headache alone.
- Lumbar punctures are not typically recommended for treatment of headache in IIH (see *7. What is the role of serial lumbar punctures in IIH?* in table 3).

##### Uncertainty

There is no evidence to support the optimal managing of headache in acute IIH.

#### Q12 What is the best approach for long-term headache management in IIH?

The pattern of headache in IIH often changes over time and needs careful assessment. There is frequently a mixed headache phenotype: headache attributed to IIH, migraine, medication overuse headache, tension-type headache, headache attributed to low CSF pressure and headache attributed to iatrogenic Chiari malformation secondary to CSF shunting.^52 53^

- A multidisciplinary team approach could be considered including, ideally, an assessment by an experienced clinician with an interest in headache management.
- In patients with IIH, the headache phenotype should be assessed. Headache therapies should be tailored to the headache phenotype.
- IIH patients with headache need clear explanation of how their headaches change over time and how to minimise the risks of developing medication overuse headache.
- Early introduction of preventative medications (migraine preventatives) should be considered as these can take 3–4 months to reach maximal efficacy.

##### Uncertainty

There is no evidence to support the optimal managing of headache in established IIH.

#### Q13 What therapeutic strategies are useful for headache in IIH?

Migrainous phenotype is noted in 68% of IIH patients with headache.^54^ Despite the lack of clinical trials, the use of migraine therapies in IIH patients with migraine headaches may be useful. Headaches with migrainous features include moderate to severe pain that maybe throbbing with photophobia, phonophobia, nausea and movement intolerance.

- Migraine attacks may benefit from triptan acute therapy used in combination with either a NSAID or paracetamol and an antiemetic with prokinetic properties.^51^ Their use should be limited to 2 days per week or a maximum of 10 days per month.^55 56^
- Migraine preventative strategies could also be tried. These are most likely to be effective in those in whom the ICP is settling and also in those whom the papilloedema has resolved (IIH in ocular remission).
- National Institute of Health and Clinical Excellence guidelines for migraine prevention therapy is useful.^51^
- Caution must be observed before selecting drugs that could increase weight (beta blockers, tricyclic antidepressants, sodium valproate, pizotifen and flunarizine) or those that could exacerbate depression, a frequent comorbidity in IIH (beta blockers, topiramate and flunarizine).
- Topiramate (see *10. Are there other drugs that are helpful in IIH?* in table 3) may help with weight loss by suppressing appetite and have an effect on reducing ICP through carbonic anhydrase inhibition. Patients need to be cautioned about potential side effects of depression, cognitive slowing, reduction of the efficacy of the contraceptive pill/oral contraceptives and potential of teratogenic effects.
- Where topiramate has excessive side effects, zonisamide may be an alternative.^49^
- In patients with migraine, candesartan can be a useful alternative to a beta blocker due to its lack of weight gain and depressive side effects.^57^ Alternatively, venlafaxine is weight neutral and helpful with depression symptoms.^58^
- Botulinum toxin A may be useful in those with coexisting chronic migraine^59^; there are no studies of botulinum toxin A in IIH.
- As with treatment of migraine, preventative drugs need to be started slowly and increased to a therapeutic tolerated dose for 3 months to enable a therapeutic trial.
- Similar to the treatment of migraine, many of these drugs are used off label in IIH.
- Lifestyle advice should be given with all headache disorders, as these can have considerable impact on the disease course. Strategies should be implemented to limit caffeine intake. Ensure regular meals and adequate hydration, exercise programme and sleep hygiene. Behavioural and stress management techniques can be implemented such as yoga, cognitive–behavioural therapy and mindfulness.

##### Uncertainty

There are no clinical trials as yet in the treatment of headache alone in IIH.

#### Q14 How should medication overuse be approached?

Medication overuse is a common issue for patients with IIH.^11^ Successfully removing excessive analgesic use significantly improves headaches.^60^ Additionally, if not addressed, MOH may prevent the optimisation and effectiveness of preventative treatments.

- Non-opioids and triptan medications may be stopped abruptly or weaned down within a month.^60^
- Opioid medications should be gradually removed, with at least 1 month painkiller free to determine effectiveness^61^

##### Uncertainty

The most effective strategies to facilitate acute analgesic medication withdrawal are not fully established.^56^

#### Q15 Should CSF diversion surgery be used in patients with IIH with headache alone?

Where papilloedema has resolved, typically, the ICP will be normalising, and conservative treatment strategies should be employed. CSF shunting to exclusively treat headache in IIH has limited evidence. Following CSF diversion 68% will continue to have headaches at 6 months and 79% by 2 years.^36^ Twenty-eight per cent can develop iatrogenic low pressure headaches,^36^ although this figure will vary depending on shunt and valve type.

- CSF diversion is generally not recommended as a treatment for headache alone in IIH.
- CSF diversion procedures for the management of headaches should only be carried out in a multidisciplinary setting and following a period of intracranial pressure monitoring.

##### Uncertainty

Patients with IIH often have coexisting migrainous headaches superimposed on the headaches secondary to raised intracranial pressure. Failure to optimise the ICP may render the migrainous headache difficult to treat.

#### Q16 Should neurovascular stenting be used in patients with IIH with headache alone?

The literature detailing stenting typically does not clearly separate the cohorts of IIH into those with visual loss, those with headaches alone and those with both. They typically also do not separate those with acute IIH, those with chronic IIH and those with IIH in ocular remission. Another major limitation is that case series are non-randomised; typically, they do not detail morphological stenosis type; they tend to be small in size with selection bias, and there is a lack of long-term follow-up.^62^

- Neurovascular stenting is not currently a treatment for headache in IIH.

##### Uncertainty

Patients with IIH often develop migrainous headaches superimposed on the headaches secondary to raised intracranial pressure. While CSF diversion procedures have not been shown to be effective for the management of headaches, this may be attributable to the migrainous component not being optimally addressed. Conversely, failure to optimise the ICP (with a CSF diversion procedures) may render the migrainous headache difficult to treat. CSF diversion procedures for the management of headaches should only be carried out in a multidisciplinary setting and following a period of intracranial pressure monitoring.

### Management of headaches in the shunted patient with IIH

Shunted patients with IIH may have significant headache morbidity, and shunt failures and overdrainage should always be considered. Understanding the underlying causes may guide management. Shunt revision should not routinely be undertaken unless there is papilloedema and a risk of visual deterioration. Many of these patients may be in ocular remission, as with chronic IIH headaches, conservative management with migraine therapies and treatment of medication overuse should be tried initially. Patients may need assessment by a experienced clinician who routinely manage headache. Medication refractory patients should be managed in a specialist headache service and discussed within a multidisciplinary setting for consideration of ICP monitoring.

#### Q17 How should an acute exacerbation of headache be investigated in those who are already shunted? (figure 4)

- For all shunted patient with IIH presenting with an acute exacerbation of headaches, funduscopy is mandatory to establish if papilloedema exists and where visual function (including formal visual fields) is documented to be worsening, then surgical intervention may be required. For those with atrophic optic nerves further care should be taken to establish whether the headache is secondary to raised intracranial pressure.
- In those where there is suspicion of infection that may be worsening the headache, CSF should be obtained for microbiological evaluation and any underlying resultant infection appropriately treated.
- A diagnostic lumbar puncture should not be routinely performed in the absence of papilloedema (unless suspicion of infection, see above)
- In those with papilloedema, some may choose to perform a diagnostic LP. This may be helpful to establish ICP level and may have implications for management choices.
- CT imaging and shunt X-ray series should not routinely be considered for those without evidence of papilloedema, as these investigations do not alter management.^63 64^
- In some ICP, monitoring may be useful.

**Figure 4:**
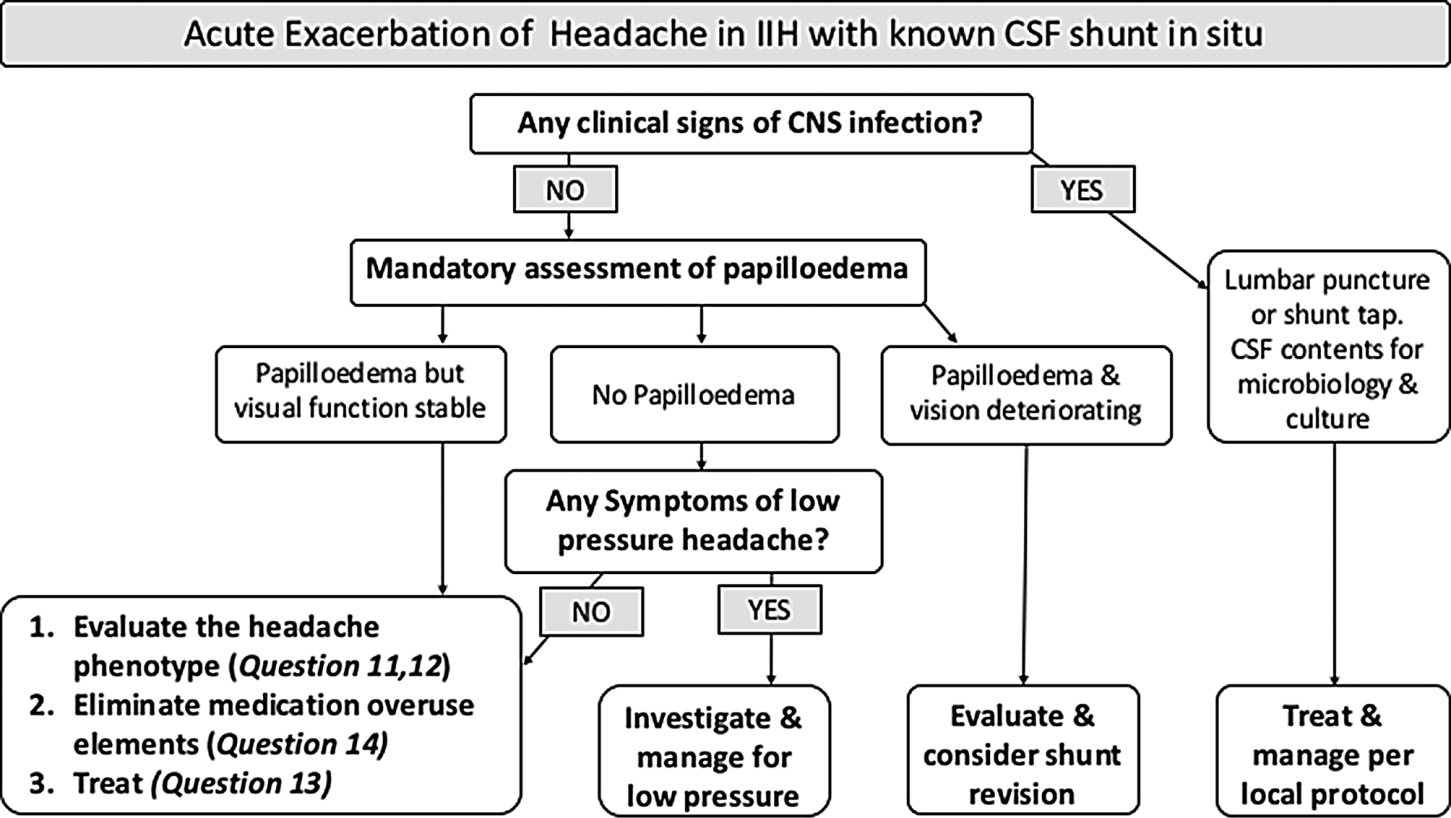
Flow chart of acute exacerbation of headache in IIH with known CSF shunt in situ. CSF, cerebrospinal fluid; IIH, idiopathic intracranial hypertension.

#### Q18 How should an acute exacerbation of headache be treated in those who are already shunted? (figure 4)

- For patients without current papilloedema or imminent risk to vision, shunt revision is not recommended.
- In shunted patients with deteriorating headaches, low pressure headache and shunt over drainage should be considered.
- In established overdrainage or low CSF pressure, consideration should be given to the valve settings or tying the shunt off.
- In the absence of shunt over drainage headache management should follow the section above (see *13. What therapeutic strategies are useful for headache in IIH?* in table 3 and figure 4: Manging acute exacerbation of headache in IIH).
- Consider medication overuse headache as a cause of acute exacerbation in shunted patients.^65^

### Clinical care

#### Q 19 Are there any other chronic problems that need to be addressed in IIH?

- All of these patients require recognition that they have been diagnosed with a rare disease and need appropriate support to deal with the psychological burden of living with a chronic condition.
- The patient with IIH may have significantly higher levels of anxiety and depression and a lower quality of life.^9 45 66^ This may be as a response to chronic pain. This needs recognition and appropriate management.
- Sleep apnoea is frequently reported in this group,^67^ and referral to respiratory service may be appropriate.
- Polycystic ovary syndrome may coexist.^68^
- Cognitive dysfunction may coexist.^69^

### Managing IIH in pregnancy

#### Q20 What advice should be given regarding drug treatments in the pregnant patients with IIH?

- A clear risk−benefit assessment regarding the necessity of acetazolamide treatment during pregnancy should be discussed with the patient as perinatal exposure in rodents has reported teratogenic effects.^70 71^
- With the limited evidence, it is difficult to make any safe recommendations on using acetazolamide during pregnancy and its manufacturers do not recommend it use.^72^
- Topiramate should not be used in pregnancy. There is clear evidence of a higher rate of fetal abnormalities following its use.^73^
- If a patient on topiramate becomes pregnant, they should reduce and discontinue it as soon as possible in line with manufacturers recommendations.
- A clear risk−benefit assessment regarding the necessity of headache treatment during pregnancy should be discussed with the patient as many of the regularly used headache medications are not recommended in pregnancy.

#### Q21 What additional considerations for management are there in the pregnant patient with IIH?

- Multidisciplinary communication among relevant experienced clinicians should occur throughout pregnancy, peridelivery and in the postpartum period.
- No specific mode of delivery should be suggested based on the fact there is a previous diagnosis of IIH.
- If not already under a weight management programme, consider referral to a weight service, so that weight gain is appropriate for gestational age of fetus as described by the American College of Obstetricians and Gynaecologists 2013 Guidelines.^74^
- Increased outpatient observation may be helpful to reassure other healthcare professionals and patients during this period.
- How should an acute exacerbation of IIH, with imminent risk to vision be managed in pregnancy?
- If the IIH is active with imminent risk of vision loss, then some would consider serial lumbar punctures as a temporising measure only until longer term measures, such as CSF diversion or ONSF, can be implemented.
- Those with imminent risk of vision loss at time of delivery should be managed in a specialist centre.

### IIH without papilloedema

#### Q22 How should IIHWOP be managed?

In patients with IIHWOP, risk of vision loss has not been identified and does not seem to develop over the disease course. Visual phenomenon such as photopsia, diplopia (from sixth nerve palsy) and functional visual field loss are common.^75^

Headache is the principal morbidity in these patients.

- Once definite IIHWOP is diagnosed, all patients should be managed as typical IIH and counselled about weight management (see *2. What is the best way to modify the disease to induce remission?* in table 3).
- Management of headache should be the same as typical IIH (see: Third principle of IIH management: reduce headache disability).
- Surgical management to control elevated intracranial pressures in IIHWOP should not routinely be considered unless advised by experienced clinicians within the multidisciplinary team setting.

### Follow-up and monitoring of IIH

#### Q23 How should we follow-up and monitor these patients?

Any patient with papilloedema should have the following documented^24^:

1. visual acuity
2. pupil examination
3. formal visual field assessment
4. dilated fundal examination to grade the papilloedema.
5. BMI calculation.
  - Formal documentation of the optic nerve head appearance, such as serial photographs or OCT imaging, is useful. There are increasing reports of the utility of transorbital ultrasound to measure optic nerve sheath diameter; however, there are considerable differences across studies on the cut-off values used as well as the efficacy of ultrasound to predict ICP.^76^
  - All patients with or without papilloedema should have an assessment of their headache to include the features of the headache/s (to aide characterisation of the headache), headache frequency and severity and frequency of analgesic use.
  - A validated headache disability score such as HIT 6 may be useful.
  - Recommendations for follow-up intervals is seen at table 4. Should there be worsening of the visual fields or papilloedema, then outpatient review should be expedited.

**Table 4:** Consensus of follow-up intervals for patients with idiopathic intracranial hypertension (IIH) based on their papilloedema grade and their visual field status

Papilloedema grade	Normal	Visual field status		
		Affected but improving	Affected but stable	Affected but worsening
Atrophic			4–6 months	Within 4 weeks
Mild	6 months	3–6 months	3–4 months	Within 4 weeks
Moderate	3–4 months	1–3 months	1–3 months	Within 2 weeks
Severe		1–3 months	Within 4 weeks	With 1 week

Note: Once papillodema has resolved, visual monitoring within the hospital services may no longer be required. However, caution in those patients who were asymptomatic at presentation, as they will likely be asymptomatic if a recurrence occurs and longer term follow-up, may need to be considered.

## Closing statement

In collaboration with many different experienced clinicians, professions and patient representatives, we have developed guidance statements for the investigation and management of adult IIH. We recognise that we were limited by the lack of high-quality evidence for the majority of the statements made and that a consensus-based approach could give authority to singular opinion. With a view to mitigate this, we have sought international expert review (GTL, RHJ and KD) and review by professional bodies (ABN, BASH, RCOphth and SBNS). Following review, a few points were upheld by the SIG such as the definition of typical IIH being diagnosed as obese and not just overweight (BMI >25 kg/m^2^), which differs from current published diagnostic criteria.^4^ As this document is aimed at a wide audience including non-IIH specialist, we wished to create criteria whereby the majority of patients with IIH would be correctly diagnosed. Hence, we needed to emphasise those patients in whom uncertainty could exist and referral to an experienced clinician maybe required. It was also highlighted that compared with our European and North American colleagues, there are few centres in the UK that perform ONSF for IIH.

These statements are not mandatory recommendations but are intended to be used as a guide for doctors who investigate and treat IIH. Despite the limitations of consensus-based methods, these statements reflect an up-to-date consensus to guide the clinician and serve our patients. Quality prospective research is required for all areas of uncertainties highlighted in this document to improve clinical outcomes for our patients with IIH.
