# Supplementary material for: Idiopathic intracranial hypertension: consensus guidelines on management
Source: J Neurol Neurosurg Psychiatry. 2018 Jun 14;89(10):1088–100. doi: 10.1136/jnnp-2017-317440 (PMC6166610; doi:10.1136/jnnp-2017-317440)
Supplement: Supplementary file 2 [file jnnp-2017-317440supp002.pdf]

AGREE appendix for IIH consensus statement:

1. **What is the overall objective for the guideline?**

   A. Improve recognition of IIH among clinicians

   B. Establish clear consensus regarding best practice for investigation and management of IIH

2. **What are the specific questions?**

   A. What criteria do we accept for diagnosis of IIH

   B. How do we investigate Papilloedema

   C. For Management the following questions were formulated:

	Principle One: Treat the underlying disease
1	*What is the best way to modify the underlying disease to induce remission?*
	Principle Two: Protect the vision
2	*How should IIH be treated when there is imminent risk of visual loss?*
3	*What is currently the best surgical procedure for visual loss in IIH?*
4	*What other surgical procedures are performed for visual loss IIH?*
5	*What is the current role of neurovascular stenting in acute IIH to prevent loss of vision?*
6	*What is the role of serial lumbar punctures in IIH?*
7	*What is the best drug treatment for IIH symptoms?*
8	*How should acetazolamide be prescribed?*
9	*Are there other drugs that are helpful in IIH?*

	**Principle Three: Manage the headache**
10	*What is the best way to manage headaches in acute IIH? (figure 4)*
11	*What is the best way to approach to long term headaches management in IIH?*
12	*How should medication overuse headache be approached?*
13	*What therapeutic strategies are useful for headache in IIH?*
14	*Should CSF diversion surgery be used in IIH patients with headache alone?*
15	*Should neurovascular stenting be used in IIH patients with headache alone?*
16	*How should an acute exacerbation of headache be investigated in those who are already shunted?*
17	*How should an acute exacerbation of headache be treated in those who are already shunted?*
	Clinical follow-up
18	*How should we follow-up and monitor these patients?*
	Special circumstances
19	*How should IIH be managed in pregnancy?*
20	*How should IIHWOP be managed?*

3. What is the population to whom the guideline is meant to apply?

The population this applies to is Adults with Typical and atypical IIH, IIH without papilloedema as defined within the document at the Definitions.

4. What groups are represented in the guideline development group?

Patient representatives (IIH UK, National charity for IIH), Neurologists, Ophthalmologists, Neurosurgeons, Nurses with a specialist interest in headache and Neuroradiologists.

5. Are all relevant groups represented?

Yes. Primary care and Emergency medicine were not included as the condition is rare and typically is treated in secondary care and above services.

6. Have the views of the target population (patients or patient groups) been sought?

Yes, IIH UK was represented at all meetings and participated fully.

6. **Who are the target users of the guidelines?**

Clinicians who diagnose and investigate papilloedema and clinicians who manage raised intracranial pressure.

7. Were methods to search for evidence systematic?

Yes

8. **What were the criteria for selecting evidence?**

Search terms included: Systemic literature review.

9. **Were strengths and limitations of evidence described?**

Yes, at each question we have a paragraph labelled uncertainties.

10. **What methods were used to formulate recommendations?**

Modified Delphi approach, as controlled data in this disease group is not readily available.

11. **Were health benefits, side effects and risks considered in formulating recommendations?**

Yes

12. **Is there an explicit link between recommendations and supporting evidence?**

Yes as referenced.

13. **Has the guideline been reviewed by experts prior to publication?**

Yes. A group of international peer colleagues have been sent the document for comment and their opinions reflected in the document.

14. **Is there a plan for updating the guideline?**

Yes

15. **Are recommendations specific and unambiguous?**

Yes

16. **Are management options clearly presented?**

Yes

17. **Are key recommendations easily identifiable?**

Yes

18. **Were facilitators or barriers to the application considered?**

To ensure the guidelines was not biased, the document was sent to experts in Europe and US and their comments reflected.

19. **Do you describe how the guidelines should be put into practice?**

Yes

20. **Have the resource implications of the recommendations been considered?**

No

21. **Do the guidelines present audit or monitoring criteria?**

Yes

22. **Have the views of the funding organisation influenced the content of the guideline?**

There is no funding. Patients and Clinicians kindly gave up their time to work on this statement.

23. **Are the competing interests of the guideline development group been recorded?**

Yes, there are no competing interests.
