# Supplementary material for: Idiopathic intracranial hypertension: consensus guidelines on management
Source: J Neurol Neurosurg Psychiatry. 2018 Jun 14;89(10):1088–100. doi: 10.1136/jnnp-2017-317440 (PMC6166610; doi:10.1136/jnnp-2017-317440)
Supplement: Supplementary file 3 [file jnnp-2017-317440supp003.pdf]

**Appendix 2: Audit recommendations from consensus guidance in IIH.**

| | Quality standard |
|---|---|
| **Investigations:** | |
| Formal visual fields at identification of papilloedema | 100% |
| Lumbar puncture | 100% |
| LP OP recorded | 100% |
| Brain imaging | 100% |
| Brain imaging within 24 hours | 75% |
| Brain Venography within 24 hours | 75% |
| ALL neuroimaging within 48hours | 100% |
| **Management:** | |
| Documentation of weight management discussed with patient. | 100% |
| BMI recorded at each visit. | 100% |
| Where a drug/each drug was used, explanation of side effects documented. | 100% |
| Potential tetragenicity of relevant medications discussed. | 100% |
| Those with papilloedema, ongoing ophthalmology assessment (including formal visual fields). | 100% |
| Those with Headache, ongoing headache management. | 100% |
