# Supplementary material for: Idiopathic intracranial hypertension: consensus guidelines on management
Source: J Neurol Neurosurg Psychiatry. 2018 Jun 14;89(10):1088–100. doi: 10.1136/jnnp-2017-317440 (PMC6166610; doi:10.1136/jnnp-2017-317440)
Supplement: Supplementary file 1 [file jnnp-2017-317440supp001.pdf]

Supplementary Table 1: Differential diagnosis of Papilloedema [19];

**Pseudopapilloedema**	Congenitally anomalous discs	Small nerves that lack a physiological cup.
	Optic nerve head drusen	Globular hyaline bodies, which may be calcified.
	Hypermetropic discs	May appear crowded and elevated
	Myopic discs	Often elevated nasally and can show leakage in fundus angiography
	Myelinated nerve fibres	
**Bilateral disc swelling**	Infective	e.g. Lyme disease, cat scratch disease, and syphilis.
	Inflammatory	e.g. Optic neuritis; Uveitis; Scleritis
	Hypertensive crisis	
	Toxic	e.g. antimicrobials, immunomodulators, chemotherapeutic agents
	Metabolic	Diabetes
	Hereditary and mitochondrial disorders	e.g. Lebers Hereditary Optic Neuropathy
	Compressive	Tumours of the optic nerve (sheath meningioma or glioma); Thyroid eye disease
**Unilateral disc swelling that uncommonly occurs bilaterally**	Vascular	Non-arteritis anterior ischaemic optic neuropathy; Arteritic anterior ischaemic optic neuropathy; Central retinal vein occlusions
